# Association between winter season and risk of death from cardiovascular diseases: a study in more than half a million inpatients in Beijing, China

**DOI:** 10.1186/1471-2261-13-93

**Published:** 2013-10-30

**Authors:** Beibei Xu, Hui Liu, Naifang Su, Guilan Kong, Xiaoyuan Bao, Jiong Li, Jing Wang, Yi Li, Xiemin Ma, Jun Zhang, Guo-Pei Yu, Lueping Zhao

**Affiliations:** 1 Medical Informatics Center, Peking University, 38 Xueyuan Rd, Haidian District, 100191, Beijing, China; 2 Section for Epidemiology, Department of Public health, Aarhus University, Aarhus, Denmark; 3 Department of Hospital Management, School of Public Health, Peking University, Beijing, China; 4 Department of Hospital Administration of Peking University Health Science Center, Peking University, Beijing, China; 5 New York Medicine College, Valhalla, NY, USA; 6 Division of Public Health Sciences, Fred Hutchinson Cancer Research Center, Seattle, WA, USA

**Keywords:** Cardiovascular disease, Winter, Seasonality, Older adults, Mortality, Asian population

## Abstract

**Background:**

Seasonal associations of cardiovascular mortality have been noted in most populations of European origin years ago, but are not well evaluated in Asian populations recently.

**Methods:**

Utilizing the electronic Hospitalization Summary Reports (HSRs) from 32 top-ranked hospitals in Beijing, China, we evaluated the association between winter season and the risk of cardiovascular death among hospitalized individuals. General additive models and logistic regression models were adjusted for confounding factors.

**Results:**

Older patients who were admitted to the hospital in the winter months (January, February, November and December) had a death risk that was increased by approximately 30% to 50% (*P* < 0.01) over those who were admitted in May. However, younger patients did not seem to experience the same seasonal variations in death risk. The excess winter deaths among older patients were associated with ischemic heart disease (RR = 1.22; 95% CI 1.13 to 1.31), pulmonary heart disease (RR = 1.42; 95% CI 1.10 to 1.83), cardiac arrhythmias (RR = 1.67; 95% CI 1.36 to 2.05), heart failure (RR = 1.30; 95% CI 1.09 to 1.54), ischemic stroke (RR = 1.30; 95% CI 1.17 to 1.43), and other cerebrovascular diseases (RR = 1.78; 95% CI 1.40 to 2.25). The risks of mortality were higher in winter months than in the month of May, regardless of the presence or absence of respiratory disease.

**Conclusions:**

Winter season was associated with a substantially increased risk of cardiovascular death among older Chinese cardiovascular inpatients.

## Background

Excess winter mortality from cardiovascular diseases has been an active research area since 1960s [1-9]. Some studies have concluded that the increase in winter deaths results from cold indoor and outdoor temperatures, while others posited that the increased winter mortality may be associated with epidemics of respiratory syncytial virus and influenza [10-15]. Studies are difficult because cardiovascular and respiratory diseases are codependent disorders and often coexist in one patient. Prior studies of seasonal variation used death certification data, which did not record disease diagnoses but recorded a primary cause of death [13-15].

Many previous studies reporting seasonal variations of cardiovascular mortality were conducted in Europe [1,4,5] and North America [2,3,6], while a few were conducted in Asia [7-9]. In those countries, excess seasonal cardiovascular mortality varies widely from 5% to 30% [16]. Moreover, excess winter mortality is reported to be relatively lower in Scandinavian countries than in the UK [17,18]. In addition to cold temperatures, the variations among countries may be related to differences in socioeconomic factors, healthcare systems, or individual lifestyles [19]. However, most previous studies of seasonal variations and health were conducted years ago, and there is little current information [1-9]. More importantly, existing studies focused on either overall or some specific cardiovascular or cerebrovascular deaths without accounting for other circulatory diseases such as pulmonary heart disease and rheumatic heart disease [1-9].

There has been a dramatic increase in the incidence of cardiovascular disease in China over recent decades [20]. Cardiovascular diseases have become the largest cause of hospitalization and mortality in China, and are expected to remain the dominant trend [20]. Due to the lack of high quality data, the association between winter season and deaths from cardiovascular disease has not been well evaluated among the Chinese population (20% of the world population). This study used data from 626,950 cardiovascular inpatients from 32 top-ranked general hospitals in Beijing to investigate whether associations between winter season and cardiovascular deaths were modified by age and cardiovascular subtypes and to determine the fraction attributable to respiratory diseases.

## Methods

### Data source and study subjects

Data were obtained from electronic Hospitalization Summary Report (HSR) in 32 top-ranked (Grade 3A) hospitals in Beijing from Jan 1, 2006 to Dec 31, 2010. Hospitals in China are ranked by the existing hospital hierarchical management approach. For both public and private hospitals, the rank is assessed according to medical services and management, quality and safety of clinical care, and technical level and efficiency. There are three grades and ten classes, and the first class hospitals are designated Grade 3A, which is the highest rank except the national special hospitals in China.

To fulfill the administrative requirements of the China Ministry of Health, every hospital in Beijing must submit an electronic HSR to a centralized health information system of the Beijing Municipal Health Bureau (full document on the official website: http://www.bjhb.gov.cn/zwfwq/ztlm/drg/ppt/201110/t20111008_41256.htm). The standard summary report is documented with basic demographics, dates of admission and discharge, pre- and post-hospitalization diagnoses, discharge status, treatments, and financial costs. This study is considered exempt since it used data collected for administrative purpose without any personal identifiers.

The HSR diagnosis was coded according to the International Classification of Diseases, 10th Revision, Clinical Modification (ICD-10-CM). The HSR contains a maximum of eight discharge diagnoses with the first diagnosis listed designated as the principal diagnosis, while the rest diagnoses were supplementary. Cardiovascular inpatients were identified if the first-listed diagnoses were cardiovascular diseases (ICD-10-CM codes I00-I99), rheumatic heart disease (codes I00-I09), hypertension (codes I10-I15), angina pectoris (codes I20), acute ischemic heart disease (codes I21-I24), chronic ischemic heart disease (code I25), pulmonary heart disease (codes I26-I28), cardiac arrhythmias (codes I44-I49), heart failure (code I50), other heart disease (codes I30-I42, I51), hemorrhagic stroke (codes I60-I62), ischemic stroke (code I63), other cerebrovascular diseases (codes I64-I62), diseases of arteries and arterioles (codes I70-I79), or other circulatory diseases (codes I80-I89, I95-I99). For this study, nonparametric estimation which requires enough sample size was used to predict the daily average mortality rate. Those aged < 30 yrs were excluded from this study for small sample size. Therefore, the final analytic sample size was 626,950. To distinguish individuals with or without respiratory diseases, cardiovascular inpatients were considered to have a respiratory disease if at least one of the supplementary diagnostic codes was J00-J47.

### Statistical analysis

Mortality rates for cardiovascular patients were calculated for each different group such that the numerator was the number of deaths for calendar-month, the winter and summer seasons, and disease subtypes. The Generalized Addictive Model (GAM), an extension of linear regression models, was used to describe average daily variations in mortality from 2006 to 2010 [21]. It models the daily average mortality rate (on a logit scale) with unspecified smooth function of admission date λ(t), which is estimated by cubic smoothing spline. The knots of the spline located at the beginning and end of each season as well as the first and last day of the five years. Using log-binomial regression models and the mortality in May as the reference, we estimated gender-, year-, and hospital-adjusted rate ratios (RRs) and 95% confidence intervals (CIs) for the other months. Analyses were performed separately for older (≥ 65 years) and younger patients (30 to < 65 years), since there is considerable heterogeneity in seasonal patterns of mortality among these two populations. To define seasons for further analysis, we grouped data of the four months of November, December, January, and February as the winter season, and the four months of May, June, July, and August as the summer season based on the results of the approximation in the risk of death between months (see Table 1). We then analyzed the associations between the winter season and the risk of cardiovascular death among older inpatients. Similar analyses were performed separately among patients with and without respiratory disease. All analyses were carried out using STATA 12.0 (StataCorp LP, College Station, Texas).

**Table 1:** Risks of death associated with hospitalization months by older and younger cardiovascular inpatients

**Month**	**≥ 65 years (n = 308,952)**				**30 to < 65 years (n = 317,998)**			
	**No.**	**Death rate (%)**	RRs (95% CI)^*^	***P*-value**	**No.**	**Death rate (%)**	RRs (95% CI)^*^	***P*-value**
January	23,912	5.0	1.49 (1.36, 1.63)	< 0.01	23,244	1.3	1.06 (0.91, 1.24)	0.43
February	23,054	4.6	1.39 (1.27, 1.52)	< 0.01	22,266	1.3	1.02 (0.87, 1.20)	0.82
March	28,587	3.5	1.06 (0.96, 1.16)	0.24	30,463	1.0	0.86 (0.74, 1.01)	0.07
April	27,745	3.9	1.18 (1.08, 1.29)	< 0.01	27,770	1.2	1.01 (0.87, 1.18)	0.91
May^†^	26,758	3.3	Reference		27,248	1.2	Reference	
June	24,555	3.5	1.03 (0.94, 1.14)	0.51	25,270	1.1	0.92 (0.78, 1.08)	0.29
July	23,689	3.7	1.10 (1.00, 1.21)	0.05	25,376	1.1	0.89 (0.76, 1.04)	0.15
August	23,695	3.7	1.09 (0.99, 1.19)	0.09	25,120	1.2	0.92 (0.78, 1.08)	0.30
September	24,213	4.0	1.19 (1.08, 1.30)	< 0.01	24,395	1.3	1.02 (0.88, 1.19)	0.79
October	28,006	4.2	1.26 (1.15, 1.37)	< 0.01	28,149	1.3	1.05 (0.90, 1.22)	0.53
November	28,027	4.3	1.29 (1.18, 1.41)	< 0.01	30,110	1.3	1.06 (0.91, 1.23)	0.45
December	26,711	4.5	1.35 (1.24, 1.48)	< 0.01	28,587	1.2	0.99 (0.85, 1.15)	0.86

^*^The rate ratios (RRs) were adjusted for gender, year, and hospital.

^†^Mortality rates in May were taken as the reference levels for comparison.

## Results

Descriptive characteristics of the study sample were shown in Table 2. Compared with younger inpatients, older inpatients had a statistically significantly higher mortality rate (4.0% vs. 1.2%, p < 0.001), longer length of hospital stay (13 days vs. 10 days, *P* < 0.001), and more supplementary diagnoses (5 diagnoses vs. 3 diagnoses, *P* < 0.001), but a lower rate of surgery (55.4% vs. 70.6%, *P* < 0.001) and a lower cost per stay (USD 2,436 vs. USD 2,583, *P* < 0.001).

**Figure 1:**
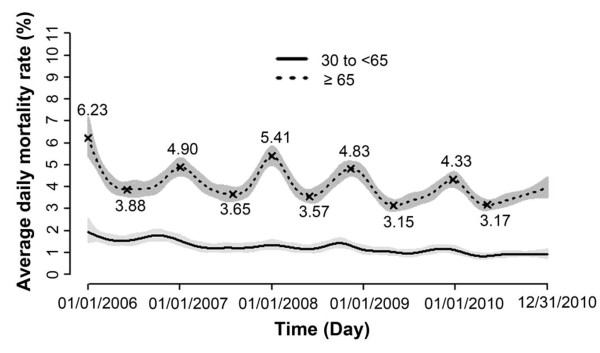
**Temporal trends of mortality rates for older (dotted line) and younger (solid line) inpatients with cardiovascular diseases (I00-I99) from 32 hospitals in Beijing, China.** The shades indicated the 95% confidence intervals. The highest and lowest mortality rate within each year were annotated above/below the dotted line. Average daily mortality rates were estimated as predictive values from the generalized additive model.

Mortality due to cardiovascular disease was presented in Figure 1. Among older patients, the mortality rate peaked during winter months and was lowest in the summer months between 2006 and 2010. Mortality rates trended downwards during both the winter (6.2% to 4.3%) and summer (3.9% to 3.2%) over the 5 years studied. The cyclical seasonal mortality pattern found among older inpatients was not observed among younger inpatients. Similar seasonal trends and overall downward trends existed for both older and younger inpatients with heart disease and cerebrovascular disease (data not shown).

**Table 2:** Characteristics of cardiovascular inpatients by two age groups

	**≥ 65 years**	**30 to < 65 years**	**Total**
	**(n = 308,952)**	**(n = 317,998)**	**(n = 626,950)**
	**No. (%)**	**No. (%)**	**No. (%)**
Year			
2006	53,960 (17.5)	50,114 (15.8)	104,074 (16.6)
2007	57,873 (18.7)	55,898 (17.6)	113,771 (18.2)
2008	58,909 (19.1)	60,511 (19.0)	119,420 (19.1)
2009	66,031 (21.4)	71,523 (22.5)	137,554 (21.9)
2010	72,179 (23.4)	79,952 (25.1)	152,131 (24.3)
Gender			
Male	172,130 (55.7)	215,099 (67.6)	387,229 (61.8)
Female	136,822 (44.3)	102,899 (32.4)	239,721 (38.2)
No. of surgery (%)	171,027 (55.4)	224,539 (70.6)	395,566 (63.1)
No. of in-hospital death (%)	12,409 (4.0)	3,856 (1.2)	16,265 (2.6)
Median length of stay (days)^*^	13 (8, 20)	10 (6, 16)	12 (7,18)
Median no. of diagnoses^*^	5 (3, 6)	3 (1, 5)	4 (2, 6)
Median cost per stay (USD)^*^	2,436 (1,419, 6,277)	2,583 (1,266, 8,221)	2,516 (1,339, 7,358)

^*^Numbers in brackets are the 25^th^ and 75^th^ percentile.

Table 1 shows the adjusted risks of cardiovascular death associated with hospitalization months among older and younger inpatients. Older patients who were hospitalized in the winter months had a 30% to 50% increased risk of death (*P* < 0.01) than those who were hospitalized in May (the month with the lowest death rate). Compared with older inpatients, however, the risks of death associated with the winter months were not statistically significant among younger inpatients. Separate analyses of deaths from cardiovascular and cerebrovascular diseases were similar (results not shown).

To evaluate which cardiovascular diseases were associated with the increased winter deaths among older inpatients (Table 3), we examined winter and summer hospitalization groups. The rates of death in winter and summer were highest for hospitalized patients diagnosed with hemorrhagic stroke (17% and 15.5%), acute ischemic heart disease (13.6% and 11.9%), and pulmonary heart disease (11.1% and 8.2%). In addition, a significantly increased mortality risk was associated with the winter season among those patients diagnosed with ischemic heart disease (RR = 1.22; 95% CI 1.13 to 1.31), pulmonary heart disease (RR = 1.42; 95% CI 1.10 to 1.83), cardiac arrhythmias (RR = 1.67; 95% CI 1.36 to 2.05), heart failure (RR = 1.30; 95% CI 1.09 to 1.54), ischemic stroke (RR = 1.30; 95% CI 1.17 to 1.43), and other cerebrovascular diseases (RR = 1.78; 95% CI 1.40 to 2.25). However, there was no positive association between winter season and deaths from hemorrhagic stroke.

**Table 3:** Associations between winter season and deaths among older cardiovascular inpatients

**Cardiovascular disease subtypes**	Winter season^*^(n = 101,704)		Summer season^*^(n = 98,748)		**RRs (95% CI)**^**†**^	***P*-value**
	**Deaths/No.**	**Death rate (%)**	**Deaths/No.**	**Death rate (%)**		
Ischemic heart disease (I20-I25)	1,826/42,761	4.3	1,358/38,946	3.5	1.22 (1.13, 1.31)	< 0.01
Angina pectoris (I20)	145/19,367	0.8	113/17,653	0.6	1.16 (0.90, 1.48)	0.25
Acute ischemic heart disease (I21-I24)	1,274/9,342	13.6	942/7,893	11.9	1.14 (1.04,1.25)	0.01
Chronic ischemic heart disease (I25)	407/14,052	2.9	303/13,400	2.3	1.28 (1.10, 1.49)	< 0.01
Pulmonary heart disease (I26-I28)	194/1,753	11.1	121/1,477	8.2	1.42 (1.10, 1.83)	< 0.01
Cardiac arrhythmias (I44-I49)	248/7,221	3.4	160/7,737	2.1	1.67 (1.36, 2.05)	< 0.01
Heart failure (I50)	372/4,454	8.4	243/3,755	6.5	1.30 (1.09, 1.54)	< 0.01
Cerebrovascular disease (I60-I69)	1,691/27,829	6.1	1,328/28,975	4.6	1.35 (1.25, 1.45)	< 0.01
Hemorrhagic stroke (I60-I62)	575/3,378	17.0	439/2,825	15.5	1.12 (0.98, 1.29)	0.10
Ischemic stroke (I63)	924/18,647	5.0	768/19,721	3.9	1.30 (1.17, 1.43)	< 0.01
Other cerebrovascular diseases (I64-I69)	192/5,804	3.3	121/6,429	1.9	1.78 (1.40, 2.25)	< 0.01
Rheumatic heart disease (I00-I09)	63/979	6.4	57/1,032	5.5	1.17 (0.80, 1.71)	0.42
Hypertensive diseases (I10-I15)	35/8,389	0.4	34/7,889	0.4	1.07 (0.66, 1.73)	0.78
Other heart disease (I30-I42, I51, I52)	73/1,587	4.6	72/1,648	4.4	1.03 (0.74, 1.45)	0.85
Diseases of arteries and arterioles (I70-I79)	139/3,969	3.5	114/4,024	2.8	1.22 (0.94, 1.57)	0.13
Other circulatory diseases (I80-I89, I95-I99)	21/2,762	0.8	16/3,214	0.5	1.47 (0.76, 2.86)	0.26

^*^Winter season consisted of January, February, November, and December, while summer season consisted of May, June, July, and August. Mortality rate in summer season was taken as the reference levels.

^†^The rate ratios (RRs) were adjusted for gender, year, and hospital.

Table 4 listed the risks of mortality associated with hospitalization months for older cardiovascular inpatients with and without respiratory disease. The mortality risks were higher in the winter months than in the month of May, regardless of whether they had a respiratory disease. However, winter increases in deaths were nearly 20% higher among patients diagnosed with respiratory diseases (40% to 60%) than those not diagnosed with respiratory diseases (20% to 40%), and the monthly mortality rates among the former were also about twice that of the latter. The analytic results of cardiovascular subtypes after excluding patients with respiratory diseases were similar to those with the influence of respiratory disease, except for hemorrhagic stroke, which also showed increases in winter deaths (RR = 1.18; 95% CI 1.01 to 1.37; results not shown).

**Table 4:** Risks of mortality associated with hospitalization month among older cardiovascular inpatients with or without respiratory diseases in Beijing, China

**Month**	Cardiovascular inpatients with respiratory diseases^* ^**(n = 50,451)**				**Cardiovascular inpatients without respiratory diseases (n = 258,501)**			
	**Death/No.**	**Death rate (%)**	**RRs (95% CI)**^**†**^	***P*-value**	**Death/No.**	**Death rate (%)**	**RRs (95% CI)**^**†**^	***P*-value**
January	355/4492	7.9	1.54 (1.28, 1.85)	< 0.01	843/19420	4.3	1.44 (1.30, 1.60)	< 0.01
February	311/3865	8.1	1.59 (1.32, 1.92)	< 0.01	757/19189	3.9	1.32 (1.19, 1.47)	< 0.01
March	266/4583	5.8	1.14 (0.94, 1.37)	0.20	743/24004	3.1	1.03 (0.93, 1.15)	0.55
April	302/4437	6.8	1.33 (1.11, 1.61)	< 0.01	787/23308	3.4	1.13 (1.02, 1.25)	0.02
May‡	210/4089	5.1	Reference		680/22669	3.0	Reference	
June	211/3661	5.8	1.07 (0.87, 1.31)	0.54	636/20894	3.0	1.03 (0.92, 1.15)	0.61
July	233/3736	6.2	1.20 (0.99, 1.47)	0.07	652/19953	3.3	1.07 (0.96, 1.19)	0.21
August	242/3873	6.3	1.16 (0.95, 1.41)	0.14	639/19822	3.2	1.06 (0.95, 1.19)	0.27
September	241/3864	6.2	1.22 (1.01, 1.49)	0.04	733/20349	3.6	1.18 (1.06, 1.31)	< 0.01
October	309/4490	6.9	1.34 (1.11, 1.62)	< 0.01	863/23516	3.7	1.23 (1.11, 1.36)	< 0.01
November	337/4662	7.2	1.43 (1.19, 1.72)	< 0.01	861/23365	3.7	1.24 (1.12, 1.38)	< 0.01
December	332/4699	7.1	1.34 (1.11, 1.61)	< 0.01	866/22012	3.9	1.34 (1.21, 1.49)	< 0.01

^*^Cardiovascular inpatients with respiratory diseases were those with a secondary diagnosis as acute upper respiratory infections, influenza and pneumonia, other acute lower respiratory infections, other diseases of upper respiratory tract, chronic lower respiratory diseases, and those with primary diagnosis or secondary diagnosis as pulmonary heart disease.

^†^The rate ratios (RRs) were adjusted for gender, year, and hospital.

‡Mortality rates in May were taken as the reference levels.

## Discussion

Our study demonstrated that overall cardiovascular mortality increased by approximately 30% to 50% during winter among cardiovascular inpatients aged ≥ 65 yrs in Beijing, China. The winter increase in mortality was seen among those with diverse subtypes of cardiovascular diseases, including ischemic heart diseases, pulmonary heart disease, cardiac arrhythmias, heart failure, and stroke. It should be emphasized that our estimates were independent of the impact of concurrent diagnosis of respiratory diseases. Results for younger patients, however, did not show a statistically significant elevated risk of cardiovascular deaths during the winter months.

Despite improved health care quality in recent years, mortality continues to peak during the winter months among older cardiovascular inpatients. Although the reason for this peak is not fully understood, the winter increase in mortality may be reduced or prevented by adequate clinical interventions since recognized triggers such as cold temperatures and respiratory infections can be effectively remediated during hospitalization.

Winter cardiovascular mortality among older inpatients is higher in Beijing than in other regions of the world. A previous study reported that excess winter mortality from coronary events was about 10% in 21 countries [22]. In addition, studies in the U.S. and in Canada also found that the difference in winter-summer mortality was about 10% for myocardial infarction, 20% for stroke, and 17% for sudden cardiac death [23,24]. The magnitude of excess winter mortality from cardiovascular disease was also found to be 28% and 48% higher in Norway and Ireland, respectively [17]. The winter peak in mortality from coronary heart disease was noted to diminish by about 2% per year from 1937 until around 1970 in the U.S., however, the trend reversed until 1991 [25]. The winter-summer difference has fluctuated in different countries, and reported data were generally based on statistics collected in the 1980s and the 1990s [22-25].

Our results showed the overall risk of death increased by 10% or more if cardiovascular inpatients aged ≥ 65 yrs were also diagnosed with a respiratory disease. However, the respiratory disease factor seemed not to contribute a large effect on the excess winter mortality, because patients diagnosed with both diseases accounted for a small fraction (about 25%) of all cardiovascular patients, and the risks of mortality were one-fold higher than those with a single cardiovascular disease in all months. Our finding is consistent with previous studies that influenza vaccination prevented only a small fraction of hospitalizations (20%) for cardiovascular diseases [26].

We also found that most of the cardiovascular disease subtypes were associated with a winter increase in mortality except for angina pectoris, rheumatic heart disease, hypertension, and arteries and arterioles diseases. For those cardiovascular subtypes with a positive association, however, the magnitudes of excess winter mortality were different, and a high difference was found for cardiac arrhythmias with the adjusted rate ratios being 1.67 (95% CI 1.36 to 2.05). The high mortality risk of cardiac arrhythmias may be associated with some abnormal activity in the cardiovascular system during winter.

The winter season may act as a surrogate variable representing other causes that ultimately lead to death. Excess winter cardiovascular mortality is principally driven by cold temperatures, and the temperature hypothesis is plausible biologically [27]. Previous studies showed that cold temperatures could increase systemic vascular resistance and fibrinogen levels, which further induced an increase in blood pressure, thrombus formation, and also enhanced fibrinolytic activity, platelet adhesiveness, and lipid levels [28-30]. Beijing is located in Northern China, with a typical cold and dry winter. Springs and autumns are both of relatively short duration. The public heating system lasts from November to March, with fairly dry and filthy air due to limited ventilation. Due to the cold winter climate, residents in Beijing tend to stay indoors in a climate-controlled condition. The large differences in the indoor/outdoor temperatures as well as longtime stay in indoor environment with poor ventilation may induce or worsen the onset of vascular conditions. Because of reduced physiologic reserves, older patients may be more vulnerable to winter stress [23]. No visible seasonal fluctuations of mortality among young patients may be due to the existence of different reasons for suffering cardiovascular diseases. Young adults suffer cardiovascular diseases because of their adverse health behaviors and job stress. Aside from cold temperatures, however, other factors, including holiday effects also increase the cardiovascular risk during the winter [27-31]. An increase in cardiovascular deaths in winter months among older patients may be associated with two important holidays - the New Year and the Chinese Spring Festival - and may have been caused by hospital factors rather than by individual holiday behaviors. During the holiday season, on-duty staffing and care may be reduced.

Our data from the 32 top-ranked hospitals in Beijing allowed us to examine seasonal associations with multiple disease endpoints and the large sample size allows statistical analysis with reliable results once potential referral biases are accounted for. The reliability of our results relates to the high quality of the HSR data. The HSR data were required by the Beijing Municipal Health Bureau for computing allocations of financial resources and evaluating hospital performance. Hospitals included in this study have a high reputation for quality in all aspects of healthcare, including diagnosis, treatment, hospital management, coding, and electronic medical record systems in China. Only inclusion of top-ranked hospitals in this study can help with eliminating quality of care as a strong potential confounding factor. However, results from this study can be only applicable to top-ranked hospitals and are not representative for all hospitals. The quality of care for other hospitals may not be as well as these top-ranked hospitals and thus can possibly have a more notably seasonal variation of daily average mortality rate.

Our study has some limitations. Despite quality controls, the HSR data are still subject to measurement errors, which include errors due to incomplete or inaccurate information recorded on summary reports as well as processing errors. It is also possible that misclassification of diseases in coding may have occurred, especially for some subtypes of cardiovascular diseases. However, the misclassification of coding, if it exists, should occur randomly and should not depend upon the seasons. The HSR data are restricted to hospitalized patients; therefore, we could not analyze cardiovascular patients who were not hospitalized—those who were not admitted to the hospital, those who died before being treated, or those who died after being discharged from the hospital. Thus, the amplitudes of excess winter mortality in our study could have been underestimated. The HSR data are abstracts describing an individual’s hospitalization, and lack clinical details that may influence the risks of death in relation to the winter season. In this study, we assumed that the standards of admission and treatment for cardiovascular diseases are the same throughout the year, and that the impact of the absence of particular clinical details is small. Finally, although we have adjusted for several confounding factors, it is possible that those residual confounding effects of other unidentified factors remain and that these could distort the positive association found between cardiovascular deaths and the winter season.

Our study has important clinical implications. We suggest that hospitals and physicians evaluate their care for older cardiovascular patients, particularly those with high-risk conditions such as respiratory infection and cardiac arrhythmias, during the winter season. The plan of care for these patients should include controlling the temperature of the wards, early identification of high-risk patients, use of preventive therapies including vascular and anti-infection treatments, sufficient health care staffing, use of adequate monitoring devices and complete discharge instructions to prevent re-admission. Clinical guidelines related to the care of older patients during the winter season may be necessary to ensure that physicians and other hospital staff are aware of the increase in cardiovascular deaths among older individuals during the winter season.

## Conclusions

Overall cardiovascular mortality increased by approximately 30% to 50% during winter among cardiovascular inpatients aged ≥ 65 yrs, while the excess winter mortality was not observed among younger cardiovascular inpatients in Beijing, China. The winter increase in mortality was seen among those with diverse subtypes of cardiovascular diseases, including ischemic heart diseases, pulmonary heart disease, cardiac arrhythmias, heart failure, and stroke. It should be emphasized that our estimates were independent of the impact of concurrent diagnosis of respiratory diseases.

## Competing interests

All authors declare that they have no competing interests.

## Authors’ contributions

BX, HL, GPY and LZ designed the research, analyzed the data, drafted the manuscript and interpreted the results. HL, NS, GLK, XYB, JW, YL, XM and JZ carried out the data management and study design. JL contributed to draft the manuscript and interpreted the results. All authors have contributed to, seen and approved the manuscript.

## Pre-publication history

The pre-publication history for this paper can be accessed here:

http://www.biomedcentral.com/1471-2261/13/93/prepub

## References

[B1] Rose G Cold weather and ischaemic heart disease Br J Prev Soc Med 1966 20 97 100 5960607 10.1136/jech.20.2.97 PMC1059032

[B2] Anderson T Le Riche W Cold weather and myocardial infarction Lancet 1970 1 291 296 4189306 10.1016/s0140-6736(70)90651-3

[B3] Rogot E Padgett S Associations of coronary and stroke mortality with temperature and snowfall in selected areas of the United States, 1962-1966 Am J Epidemiol 1976 103 565 575 937340 10.1093/oxfordjournals.aje.a112261

[B4] Haberman S Capildeo R Clifford RF The seasonal variation in mortality from cerebrovascular disease J Neurol Sci 1981 52 25 36 10.1016/0022-510X(81)90131-3 7299414

[B5] Douglas A Dunnigan M Allan T Rawles J Seasonal variation in coronary heart disease in Scotland J Epidemiol Community Health 1995 49 575 582 10.1136/jech.49.6.575 8596091 PMC1060171

[B6] Bull G Morton J Environment, temperature and death rates Age Ageing 1978 7 210 224 10.1093/ageing/7.4.210 727071

[B7] Marshall RJ Scragg R Bourke P An analysis of the seasonal variation of coronary heart disease and respiratory disease mortality in New Zealand Int J Epidemiol 1988 17 325 331 10.1093/ije/17.2.325 3403127

[B8] Enquselassie F Dobson A Alexander H Steele P Seasons, temperature and coronary disease Int J Epidemiol 1993 22 632 636 10.1093/ije/22.4.632 8225736

[B9] Pan WH Li LA Tsai MJ Temperature extremes and mortality from coronary heart disease and cerebral infarction in elderly Chinese Lancet 1995 345 353 355 10.1016/S0140-6736(95)90341-0 7845116

[B10] Clinch JP Healy JD Housing standards and excess winter mortality J Epidemiol Community Health 2000 54 719 720 10.1136/jech.54.9.719 10942456 PMC1731747

[B11] Eng H Mercer JB Seasonal variations in mortality caused by cardiovascular diseases in Norway and Ireland J Cardiovasc Risk 1998 5 89 95 10.1097/00043798-199804000-00004 9821061

[B12] The Eurowinter Group Cold exposure and winter mortality from ischaemic heart disease, cerebrovascular disease, respiratory disease, and all causes in warm and cold regions of Europe Lancet 1997 349 1341 1346 9149695

[B13] Han LL Alexander JP Anderson LJ Respiratory syncytial virus pneumonia among the elderly: an assessment of disease burden J Infect Dis 1999 179 25 30 10.1086/314567 9841818

[B14] Thompson WW Shay DK Weintraub E Brammer L Cox N Anderson LJ Fukuda K Mortality associated with influenza and respiratory syncytial virus in the United States JAMA 2003 289 179 186 10.1001/jama.289.2.179 12517228

[B15] Reichert TA Simonsen L Sharma A Pardo SA Fedson DS Miller MA Influenza and the winter increase in mortality in the United States, 1959-1999 Am J Epidemiol 2004 160 492 502 10.1093/aje/kwh227 15321847

[B16] Curwen M Excess winter mortality: a British phenomenon? Health Trends 1991 22 169 175

[B17] Mercer JB Cold–an underrated risk factor for health Environ Res 2003 92 8 13 10.1016/S0013-9351(02)00009-9 12706750

[B18] Laake K Sverre JM Winter excess mortality: a comparison between Norway and England plus Wales Age Ageing 1996 25 343 348 10.1093/ageing/25.5.343 8921136

[B19] Healy JD Excess winter mortality in Europe: a cross country analysis identifying key risk factors J Epidemiol Community Health 2003 57 784 789 10.1136/jech.57.10.784 14573581 PMC1732295

[B20] Yang G Kong L Zhao W Wan X Zhai Y Chen LC Koplan JP Emergence of chronic non-communicable diseases in China Lancet 2008 372 1697 1705 10.1016/S0140-6736(08)61366-5 18930526

[B21] Hastie T Tibshirani R General addictive models: some applications J Am Stat Assoc 1987 82 371 386 10.1080/01621459.1987.10478440

[B22] Barnett AG Dobson AJ McElduff P Salomaa V Kuulasmaa K Sans S Cold periods and coronary events: an analysis of populations worldwide J Epidemiol Community Health 2005 59 551 557 10.1136/jech.2004.028514 15965137 PMC1757082

[B23] Sheth T Nair C Muller J Yusuf S Increased winter mortality from acute myocardial infarction and stroke: the effect of age J Am Coll Cardiol 1999 33 1916 1919 10.1016/S0735-1097(99)00137-0 10362193

[B24] Gerber Y Jacobsen SJ Killian JM Weston SA Roger VL Seasonality and daily weather conditions in relation to myocardial infarction and sudden cardiac death in Olmsted County, Minnesota, 1979 to 2002 J Am Coll Cardiol 2006 48 287 292 10.1016/j.jacc.2006.02.065 16843177

[B25] Seretakis D Lagiou P Lipworth L Signorello LB Rothman KJ Trichopoulos D Changing seasonality of mortality from coronary heart disease JAMA 1997 278 1012 1014 10.1001/jama.1997.03550120072036 9307350

[B26] Nichol KL Nordin J Mullooly J Lask R Fillbrandt K Iwane M Influenza vaccination and reduction in hospitalizations for cardiac disease and stroke among the elderly N Engl J Med 2003 348 1322 1332 10.1056/NEJMoa025028 12672859

[B27] Kloner RA Natural and unnatural triggers of myocardial infarction Prog Cardiovasc Dis 2006 48 285 300 10.1016/j.pcad.2005.07.001 16517249

[B28] Argiles A Mourad G Mion C Seasonal changes in blood pressure in patients with end-stage renal disease treated with hemodialysis N Engl J Med 1998 339 1364 1370 10.1056/NEJM199811053391904 9801397

[B29] Neild PJ Syndercombe-Court D Keatinge WR Donaldson GC Mattock M Caunce M Cold-induced increases in erythrocyte count, plasma cholesterol and plasma fibrinogen of elderly people without a comparable rise in protein C or factor X Clin Sci (Lond) 1994 86 43 48 8306550 10.1042/cs0860043

[B30] Keatinge WR Coleshaw SR Cotter F Mattock M Murphy M Chelliah R Increases in platelet and red cell counts, blood viscosity, and arterial pressure during mild surface cooling: factors in mortality from coronary and cerebral thrombosis in winter Br Med J (Clin Res Ed) 1984 289 1405 1408 10.1136/bmj.289.6456.1405 6437575 PMC1443679

[B31] Phillips DP Jarvinen JR Abramson IS Phillips RR Circulation 2004 110 3781 3788 10.1161/01.CIR.0000151424.02045.F7 15596560

